# CANOPTIPHYS study protocol: Optimising PHYSical function before CANcer surgery: effects of pre-operative optimisation on complications and physical function after gastrointestinal cancer surgery in older people at risk—a multicentre, randomised, parallel-group study

**DOI:** 10.1186/s13063-022-07026-w

**Published:** 2023-01-19

**Authors:** Mikael Andersson, Monika Egenvall, Johanna Danielsson, Anders Thorell, Christian Sturesson, Mattias Soop, Malin Nygren-Bonnier, Elisabeth Rydwik

**Affiliations:** 1 Department of Neurobiology, Care Sciences and Society, Division of Physiotherapy, Karolinska Institutet, Stockholm, Sweden; 2 Department of Molecular Medicine and Surgery, Karolinska Institutet, Stockholm, Sweden; 3 Department of Pelvic Cancer, Colorectal Surgery Unit, Karolinska University Hospital, Stockholm, Sweden; 4 Department of Clinical Sciences, Danderyd Hospital, Karolinska Institutet, Stockholm, Sweden; 5 Department of Surgery, Ersta Hospital, Stockholm, Sweden; 6 Division of Surgery, Department of Clinical Science, Intervention and Technology (CLINTEC), Karolinska Institutet and Karolinska University Hospital, Stockholm, Sweden; 7 Theme Women’s Health and Allied Health Professionals, Medical Unit Occupational Therapy & Physiotherapy, Karolinska University Hospital, Stockholm, Sweden; 8 Stockholm Region Council, FOU nu, Research and Development Unit for the Elderly, Järfälla, Sweden

**Keywords:** Prehabilitation, Cancer surgery, Optimisation, RCT, Exercise, Post-operative complications, Disability

## Abstract

**Background:**

This multicentre study explores the effects of pre-operative exercise on physical fitness, post-operative complications, recovery, and health-related quality of life in older individuals with low pre-operative physical capacity scheduled to undergo surgery for colorectal cancer. We hypothesise that this group of patients benefit from pre-operative exercise in terms of improved pre-operative physical function and lower rates of post-operative complications after surgery compared to usual care. Standardised cancer pathways in Sweden dictate a timeframe of 14–28 days from suspicion of cancer to surgery for colorectal cancer. Therefore, an exercise programme aimed to enhance physical function in the limited timeframe requires a high-intensity and high-frequency approach.

**Methods:**

Participants will be included from four sites in Stockholm, Sweden. A total of 160 participants will be randomly assigned to intervention or control conditions. Simple randomisation (permuted block randomisation) is applied with a 1:1 allocation ratio. The intervention group will perform home-based exercises (inspiratory muscle training, aerobic exercises, and strength exercises) supervised by a physiotherapist (PT) for a minimum of 6 sessions in the pre-operative period, complemented with unsupervised exercise sessions in between PT visits. The control group will receive usual care with the addition of advice on health-enhancing physical activity. The physical activity behaviour in both groups will be monitored using an activity monitor. The primary outcomes are (1) change in physical performance (6-min walking distance) in the pre-operative period and (2) post-operative complications 30 days after surgery (based on Clavien-Dindo surgical score).

**Discussion:**

If patients achieve functional benefits by exercise in the short period before surgery, this supports the implementation of exercise training as a clinical routine. If such benefits translate into lower complication rates and better post-operative recovery or health-related quality of life is not known but would further strengthen the case for pre-operative optimisation in colorectal cancer.

**Trial registration:**

ClinicalTrials.gov NCT04878185. Registered on 7 May 2021. https://clinicaltrials.gov/ct2/home

## Administrative information

Note: the numbers in curly brackets in this protocol refer to SPIRIT checklist item numbers. The order of the items has been modified to group similar items (see http://www.equator-network.org/reporting-guidelines/spirit-2013-statement-defining-standard-protocol-items-for-clinical-trials/).

**Table:** 

Title {1}	CANOPTIPHYS study protocol: Optimising PHYSical function before CANcer surgery: effects on complications and quality of life after gastrointestinal cancer surgery in older people at risk. A multicentre, randomised, parallel-group superiority study.
Trial registration {2a and 2b}.	2a: ClinicalTrials.gov Identifier: NCT04878185 2b: Se Item 2a.
Protocol version {3}	May 7, 2021, version 1.
Funding {4}	Financial support for the study is provided through grants from the non-profit organisations The Swedish Cancer Society (period for support: 2020-2023) and The Swedish Research Council (period for support: 2020-2023). Both organisations provide support through grants aimed at salary expenses (data collection personnel at recruitment sites, doctoral- and postdoctoral positions) and costs related to material (lab equipment). None of the funding organisations has any vested interest in the outcome of the study. Financial support was also provided through The Erling-Persson Foundation (a framework funding grant to AT, Grant # 140604).
Author details {5a}	**MA, JD**: Department of Neurobiology, Care Sciences and Society, Division of Physiotherapy, Karolinska Institutet, Stockholm, Sweden. **ME**: Department of Molecular Medicine and Surgery, Karolinska Institutet, Stockholm, Sweden; Department of Pelvic Cancer, Colorectal Surgery Unit, Karolinska University Hospital, Stockholm, Sweden **AT:** Department of Clinical Sciences at Danderyds Hospital, Karolinska Institutet & Department of Surgery, Ersta Hospital, Stockholm, Sweden **CS:** Division of Surgery, Department of Clinical Science, Intervention and Technology (CLINTEC), Karolinska Institutet and Karolinska University Hospital, Stockholm, Sweden. **MS:** Department of Clinical Sciences at Danderyds Hospital, Karolinska Institutet & Department of Surgery, Ersta Hospital, Stockholm, Sweden **MN-B:** Department of Neurobiology, Care Sciences and Society, Division of Physiotherapy, Karolinska Institutet, Stockholm, Sweden; Theme Women’s Health and Allied Health Professionals, Medical Unit Occupational Therapy & Physiotherapy, Karolinska University Hospital, Stockholm, Sweden. **ER:** Department of Neurobiology, Care Sciences and Society, Division of Physiotherapy, Karolinska Institutet, Stockholm, Sweden; Theme Women’s Health and Allied Health Professionals, Medical Unit Occupational Therapy & Physiotherapy, Karolinska University Hospital, Stockholm, Sweden; Stockholm Region Council, FOU nu, Research and Development Unit for the Elderly, Järfälla, Sweden.
Name and contact information for the trial sponsor {5b}	Karolinska Institutet, 171 77 Stockholm
Role of sponsor {5c}	Study sponsors and funding parties will have no role in or authority over any aspect of the design, collection, management, analysis, and interpretation of data; writing of the report; and decisions regarding the publication process.

## Introduction

### Background and rationale {6a}

Colorectal cancer (CRC) affects about 25/100,000 people worldwide, making it the third most common type of cancer in Sweden and globally [1, 2]. The primary treatment option is surgery, with or without oncological treatment [3]. Even though incidence rates for early-onset CRC (diagnosis before age 50) are increasing, CRC remains primarily a disease of older people [4]. Hospitalisation and major surgery are associated with periods of bed rest, resulting in loss of muscle function and strength [5], which can be particularly detrimental in older people due to their often diminished functional reserve capacity [6].

Increasing age is usually accompanied by disability, multimorbidity, and frailty, which further increases the risk of surgery in older people [7]. According to Fried, the frail patient is characterised by a deficit in three of the following areas; exhaustion, weight loss, low activity, slow walking, and grip strength, but even only one affected area could characterise someone as “pre-frail” [8]. Screening for frailty prior to surgery could serve dual purposes: risk stratification to aid in the decision of surgery or to identify “treatable traits,” i.e. areas that would potentially be amendable for an intervention [9]. In the present study, slow walking speed will be used to identify frail and pre-frail CRC patients at higher risk of post-operative complications [10, 11]. After major abdominal surgery in older people, the proportion of people with protracted disabilities is between 10 and 50% at 6 months after surgery [12].

The concept of prehabilitation was proposed as an effort to counteract detrimental treatment-related effects in intensive care [13]. The goal of prehabilitation is to enhance the functional capacity (i.e. strength, endurance) of subjects prior to the known stressor of surgery or hospitalisation [13, 14]. When applied to cancer treatment, prehabilitation has been defined as “a process on the continuum of care that occurs between the time of cancer diagnosis and the beginning of acute treatment” [15]. Efforts are particularly motivated when directed towards subjects, who are considered frail, who are at increased risk of peri- and post-operative complications [16, 17]*.* In a systematic review from 2016, Bruns et al. concluded that preoperative exercise for older individuals with colorectal cancer may improve physical performance. However, this improvement does not necessarily affect morbidity and recovery since no effect on postoperative complications (PC) was demonstrated [18]. Bousquet-Dion et al. compared a tri-modular approach of moderate-intensity-, partially supervised exercise together with nutritional support, and anxiety-reduction strategies. The programme was delivered either before or after colorectal cancer surgery [19].

No significant differences on postoperative complications, walking capacity, or LOS were seen between groups despite achieving excellent adherence to the exercise regimen (90–98%). However, they observed that especially the inactive patients in the prehabilitation group were more likely to have improved their 6-min walk distance. Furthermore, they speculate that for higher gains, a higher intensity of pre-operative exercises might be needed [19]. Building on their work, they later conducted a study exploring the effect of their prehabilitation programme on postoperative complications when applied to older frail individuals. Similar to their previous results, no significant differences were detected between groups [20]. Their results could possibly be attributed to the high amount of minimal invasive surgery, or insufficient exercise dose in the limited time-frame. Adopting a moderate- to high-intensity prehabilitation programme, an almost 50% reduction of PC in a group of high-risk patients (based on low physical fitness) has been reported [21]. The intervention consisted of a 3-week community-based, supervised exercise programme. A similar reduction of the risk of PC (50%) was seen in another study who randomised high-risk patients into either usual care or a 4-week prehabilitation programme [22]. Besides prehabilitation protocols targeting aerobic exercises and general strength training, the inclusion of inspiratory muscle training (IMT) seems to convey added benefits [23, 24]. One study conclude that a period of at least 14 days, preferably supervised sessions and in addition to other exercises, is an effective method to reduce LOS [25] and postoperative pulmonary complications. In contrast, Dronkers et al. found benefits only for respiratory muscle function, not functional tasks, physical activity, or LOS [24]. The heterogeneity of study designs, sample characteristics, and outcomes all make conclusions regarding the effects of prehabilitation uncertain, and more rigorous standardisation is warranted.

Based on previous studies in the prehabilitation field that focus on the physical aspects of optimisation, we conclude that an effective prehabilitation programme in CRC should preferably include the following components: a high-intensity approach of at least 2 weeks’ duration [23], incorporation of both IMT and whole body exercises [23], target subjects of increased risk of adverse outcomes [21, 22], and preferably be home-based with support from a professional to improve adherence to training [26]. Outcome measures in the older oncological patient should furthermore capture both direct effects such as survival and include measures on quality of life, dependency, and ADL-tasks [27].

## Objectives {7}

We hypothesise that older CRC patients with low physical capacity will benefit from high-intensity physical exercises, both in a short perspective (increased physical capacity preoperatively) and in a longer view (lower risk for PC and better recovery after discharge from hospital), when compared to usual care.

### Primary objectives

To compare the effects on pre-operative physical function (6-min walk distance) and PC (Clavien-Dindo classification of surgical complications) 30 days after surgery between the high-intensity exercise group compared to usual care.

### Secondary objectives

To compare the groups regarding the following aspects:

- Change in physical function (lower extremity functional strength, inspiratory muscle strength, walking distance) at the end of the pre-operative period and at discharge
- Length of stay and destination of discharge
- Self-reported symptoms (patient-reported recovery) at discharge
- Long-term effects (6 months and 1 year post-surgery) on health-related quality of life, physical activity, activities of daily living, and patient-reported recovery.

## Trial design {8}

The CANOPTIPHYS trial is a randomised parallel group, superiority trial using permuted block randomisation with a 1:1 allocation ratio.

## Methods: participants, interventions, and outcomes

### Study setting {9}

Screening and recruitment of participants are performed at four sites: Karolinska University Hospital in Solna and Huddinge, South General Hospital, and Ersta Hospital, situated in the Stockholm city area, Sweden.

Physiotherapists (PT) from the Stockholm County home-rehabilitation units will deliver the intervention in the participants’ homes. Fifteen home-rehabilitation units have been recruited to participate in the study, chosen for maximal coverage based on geographical dispersion and for operational purposes limited to the Stockholm County area.

### Eligibility criteria {10}

Inclusion criteria (for participants):

- Awaiting surgery due to suspicion of colorectal cancer or colorectal liver metastases
- Age 65 years or older
- Maximal walking speed below 2 m/s
- Residential address in Stockholm County
- Understand and speak the Swedish language

Exclusion criteria

- Surgical procedures which hinder post-operative measurements (for instance, hyperthermic intraperitoneal chemotherapy, abdominoperineal rectal resection with musculocutaneous flap)
- If time to surgery < 14 days and postponing the operation for additional 7–14 days constitutes a medical risk (as assessed by the patient’s clinicians)

Inclusion criteria (*for sites)*

- Organisational readiness for participation (availability of vital staff: PTs, nurses, doctors)

### Who will take informed consent? {26a}

Informed consent will be obtained by trained study personnel (nurses, PTs, research nurses) at the recruitment sites. Due to organisational differences between sites, minor differences are allowed regarding the process of obtaining informed consent and stem from different scheduling of multidisciplinary team conferences and different availability of staff. However, all participants are given equal opportunity to discuss all questions and are provided with the same written information. The only difference between sites pertains to who is providing the information to the participant (PT, nurse, or physician). The broad outline of inclusion is as follows:

A nurse performs a first screening of suitable participants among referred patients based on age and residential address criteria. These participants are further discussed at the multidisciplinary team conference, where the date of surgery is scheduled to accommodate inclusion in the study. If no medical reason is identified necessitating surgery to be performed within 14 days, a final screening for suitability is done based on physical function (walking speed < 2 m/s [28]). Participants who fulfil the inclusion criteria are then approached by a research nurse and given brief information on the aims and implications of participation in the study. Written information and a consent form are provided to the subject. They are informed that a physiotherapist from the department will contact them by phone with further information and to answer any questions regarding the study. If verbal consent is given, the physiotherapist schedules the baseline visit where the written consent form is collected.

### Additional consent provisions for collection and use of participant data and biological specimens {26b}

Not applicable. No collection or use of participant data or biological specimens for ancillary studies is planned.

### Interventions

#### Explanation for the choice of comparators {6b}

Standardised Cancer care Pathways (SCP) for cancer were introduced in Sweden in 2015 to shorten waiting times, increase patient satisfaction, and reduce regional variability [29]. At present, pre-operative exercise is not included, and the usual care setting was therefore chosen as the comparator group. The control group will receive written advice to be physically active at a moderate level for at least 150 min/week following current guidelines [30]. In addition, when a participant in the control group is informed about their group allocation over telephone, general recommendations for physical activity will be repeated and discussed briefly in relation to the current activity of the participant. The physical activity levels in the entire pre-operative period (from t_0_ to t_1_) will be monitored through a thigh-mounted activity monitor.

#### Intervention description {11a}

The intervention group will receive moderate- to high-intensity physical exercise for 14–17 days before surgery. The intervention takes place in the home setting of study participants and is monitored by a PT. A minimum of six PT visits will be performed, and between PT visits, participants are instructed to perform daily unsupervised exercises (tailored to individually chosen goals.

Each daily exercise consists of three blocks of exercises targeting respiratory muscles (block I), aerobic exercises (block II), and functional strength exercises (block III). The target intensity for all exercise blocks is set at 5–7 on the Borg CR-10 scale [31]. Exercises are tailored to the specific goals of participants using the Patient-Specific Functional Scale (PSFS) [32, 33].

*Block I: Inspiratory muscle training* (IMT) performed twice daily for 30 breaths against external resistance. The Powerbreathe K3 device (POWERbreathe International Limited, Northfield Road, Southam, Warwickshire, CV47 0FG, England, UK) is used in the IMT training. The device allows for loading of the inspiratory muscles in increments of 1 cmH2O pressure (minimal load 5, maximal load 200 cmH2O). The initial load is set at 50% av the maximal inspiratory pressure generated at the baseline visit. Participants and PTs are instructed to increase the training load if participants’ rating of exercise intensity at the end of the training session is below 5 on the Borg CR-10 scale.

*Block II: Aerobic exercises* is performed for a duration of 20 min. Exercises are chosen freely by the PT with the possibility to utilise both indoor as well as outdoor activities depending on the living condition of the participants. Exercises utilising an interval training set-up are recommended to allow for periods of high-intensity exercises interspersed with periods of lower intensity.

*Block III: Functional strength training*: A few exercises (“Step-up” and “Sit-to-stand”) are considered essential exercises and are included in the exercise regimen of all participants. These essential exercises are chosen based on the possibility to standardise the increase of training intensity using provided weight belts. Besides these exercises, PTs are instructed to use their discretion in designing exercises to improve patients’ performance on the PSFS-identified activities.

#### Unsupervised exercise sessions

Between supervised exercise sessions, participants are instructed to continue with daily IMT sessions as described previously in block I as well as a selection of exercises from blocks II and III. To allow for recovery and to avoid over-exertion, some of the unsupervised sessions will be of a low-intensity type, such as a slow walk of 20–30 min. The frequency, intensity, time, and type of exercises are instructed verbally and supplemented by a descriptive brochure. The number of unsupervised sessions will vary between 3 and 4 per week.

#### Criteria for discontinuing or modifying allocated interventions {11b}

Adverse events (AE) are monitored throughout the intervention period. Should AEs occur, the intervention is stopped, and the surgeon responsible for the subject’s cancer care is contacted and decides whether the interventions should be discontinued or proceed as planned. Should participants choose to withdraw consent or discontinue for an AE, they will be retained in the study, and an intention-to-treat approach will be used in the analysis.

#### Strategies to improve adherence to interventions {11c}

Participation does not incur additional costs related to PT visits or study-related hospital visits (*t*_−1_ and *t*_1_) for which free taxi transportation is offered. Participants in the intervention group will receive personalised feedback on their activity behaviour based on information from the activPAL-monitor. Feedback is given approximately halfway through the intervention period and is focused on how activity patterns (time spent sitting and standing and transitions to the upright position, number of steps, time spent stepping) are related to recommended levels for health-enhancing physical activity. Based on the collected data, individual recommendations on what actions the subject can take to increase their health-enhancing behaviour further are formulated and conveyed to participants at the next supervised exercise session.

PT and participant adherence will be monitored in the following ways:

- An exercise protocol filled out by the PT during each supervised session with information regarding the type, duration, and/or number of repetitions performed and achieved intensity for each exercise block. Furthermore, when sufficient intensity is not achieved, the PT is asked to state the reason for this (e.g. pain or fatigue).
- A calendar filled out by the participant for each unsupervised session containing the same information as stated above.
- Automatically stored information regarding number of sessions of IMT performed which will be transferred from the IMT device to a protocol.
- The ActivPAL activity monitor will measure the level of physical activity.

Taken together, this will provide useful information about whether the intervention was delivered and performed according to the study protocols.

#### Relevant concomitant care permitted or prohibited during the trial {11d}

No restrictions are imposed for concomitant care for participants enrolled in the study.

#### Provisions for post-trial care {30}

Participants have insurance coverage according to Swedish health regulation, which states that injuries associated with participation in medical research are covered, including participants that choose to withdraw their consent. If the intervention is deemed beneficial for participants, the treatment regimen will be freely accessible to care providers through the publication of the study in a peer-reviewed journal.

### Outcomes {12}

Time points for outcome assessments are referred to by the annotations described in Table 1 (*t*_−1_ to *t*_8_).

**Table 1:**
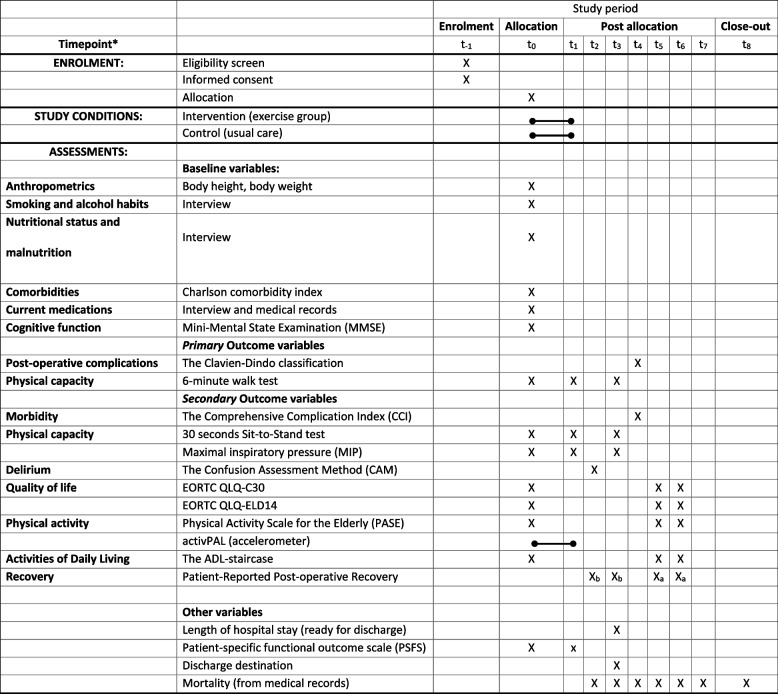
Participant timeline

*t_-1 enrolment_, t_0_ = baseline assessment and point of randomization, t_1_= admission, t_2_ = two to three days post-surgery, t_3_ = ready to discharge, t_4_ = 30 days post-surgery, t_5_ = 6-month post-surgery, t_6_ = 1-year post-surgery, t_7_ = 2 years post-surgery t_8_ = 3 years post-surgery/close-out. X_a_ = PRP original version, X_b_= PRP hospital version excluding questions 11 and 12.

#### Primary outcomes

- Pre-operative outcome: change in maximal walking distance

Difference between treatment groups regarding the change in walking distance during a 6-min walk test (6MWT) [34] during the pre-operative period. The outcome is the maximal walking distance (metres) participants can cover in 6 min. Comparisons between changes in distance between the intervention and control groups will be performed. A difference of 50 m will be considered clinically significant and was used in the power calculations for sample size.

Time points: Baseline (*t*_0_), at hospital admission (or the day before (*t*_1_), and at discharge from hospital (*t*_3_).

- Post-operative outcome: complications 30 days post-surgery

The difference in complications between groups regarding post-operative complications (PC) 30 days post-surgery according to Clavien-Dindo (CD) and any complications up to day 30 will be considered [35]. The CD grading system allows for quantifying post-operative complications based on the type of treatment needed to correct the complication. Grade I requires the least treatment; grade V is death. The number and proportion of participants with PC will be presented. No minimal important difference (MID) level for the CD system is recommended, and in the present study, a difference of 1 grade is considered important and was used in the power calculations for sample size.

Time-point: 30 days post-surgery (*t*_4_)

#### Secondary outcomes

- The Comprehensive Complication Index (CCI) [36] will be used to further describe the combined burden of PCs. The CCI is based on the CD complication and integrates information from both the number of complications and their severity and can be calculated using a freely available online calculator (https://www.assessurgery.com). The resulting metric is a continuous score ranging from 0 (no burden of complications) to 100 (death from complications). A difference of 10 points has been proposed as MID and to correspond to a 1-grade difference in the CD classification [36].

Time point: 30 days post-surgery (*t*_4_)

- Length of hospital stay

The number of days spent in the hospital will be collected from the medical records. The outcome is the number of days. Time frame: Day of hospital admission (*t*_1_) to the day the patient is considered ready to discharge (*t*_3_) (surgeons’ decision).

- Quality of life

The Swedish version of *The European Organisation for Research and Treatment of Cancer (EORTC) quality of life questionnaire core-30 (EORTC-CLQ-C30)* [37]. The scale is comprised of nine subscales: one global health status scale, five functioning scales, and three symptom scales. The scales range from 0 to 100. Higher scores for the global health status scale and functioning scales indicate better outcomes, whereas, for the symptom scales, higher scores indicate worse outcomes. MID in CRC is reported to lie between 5 and 10 points [38].

Time points: Baseline (*t*_0_), 6 months (*t*_5_), and 12 months (*t*_6_) post-surgery.

*The European Organisation for Research and Treatment of Cancer (EORTC) quality of life questionnaire for the Elderly Cancer Patients Module (EORTC QLQ-ELD14)* [39]. The scale comprises five subscales: mobility, family support, worries about the future, maintaining autonomy and purpose, and burden of illness. The scales range from 0 to 100. Higher scores indicate better outcomes for the family support and autonomy and purpose scales. For the mobility, worries, and burden of illness scales, higher scores indicate worse outcomes. Time points: Baseline (*t*_0_), 6 months (*t*_5_), and 12 months (*t*_6_) post-surgery.

- Destination of discharge from the hospital

Data on the destination of discharge will be collected from the patient records. The information will be categorised into the home, rehabilitation facility, or further care (e.g. a geriatric clinic or residential care). Time-point: at hospital discharge (*t*_3_)

- Patient-reported symptoms

The *Post-operative Recovery Profile (PRP)* [40]. The scale consists of physical symptoms, physical function, psychological and social impact, and activity. The results are divided into five categories, ranging from not recovered at all (< 7 points) to fully recovered (19 points).

A 17-question version adopted for in-hospital use was applied in the early post-operative phase (*t*_2_ and *t*_3_). In this version, questions 11 (impact on sexual life) and 12 (impact on social life) were omitted. In the long-term follow-up (*t*_5_ and *t*_6_), the original version was used. This adaptation was based on feedback from participants in the feasibility study [41] preceding this RCT that commented that those were not applicable during the hospital stay. The difference in maximal points between versions will be considered in the analysis of data. Time points: 2–3 days post-surgery (*t*_2_), day of hospital discharge (*t*_3_), 6 months (*t*_5_), and 12 months (*t*_6_) post-surgery.

- Delirium

The *Confusion Assessment Method (CAM)* [42] is an instrument consisting of 9 items. The observer scores the subject on the presence of acute onset or fluctuating course in a change in mental status (criteria 1) *and* inattention (criteria 2) with signs of either disorganised thinking (criteria 3) *or* altered level of consciousness (criteria 4). The outcome is binary, *confusion*, or *no confusion* and is based on the presence of criteria 1 *and* 2 in conjunction with criteria 3 *or* 4. Time points: 2–3 days post-surgery (*t*_2_).

- Change in lower extremity strength

*The 30-second chair stand test* [43] will be used to measure functional lower extremity strength. The outcome is the number of times the patient comes to a full standing position in 30 s. Time points: Baseline (*t*_0_), at hospital admission (or the day before) (*t*_1_), at hospital discharge (*t*_3_).

- Change in maximal inspiratory muscle strength

*Maximal voluntary inspiratory pressure* (MIP) at the mouth is measured using the MicroRPM (Micro Medical/CareFusion, Kent, UK). The MicroRPM measures the maximal pressure obtained from a maximal effort inspiration starting from the point of full expiration/residual lung volume [44]. The outcome is maximal pressure in cm H_2_O. Time points: Baseline (*t*_0_), at hospital admission (or the day before) (*t*_1_), at hospital discharge (*t*_3_).

- Level of independence in daily living

*The ADL-staircase* [45] is a hierarchical scale with ten items, including both personal and instrumental activities. The subject’s ability in these activities is graded on three levels, ranging from independent to dependent. Time points: Baseline (*t*_0_), 6 months post-surgery (*t*_5_), 12 months post-surgery (t_6_)

- Physical activity level

The *Physical Activity Scale for the Elderly* [46] includes time spent in sitting, exercise, leisure, household/gardening, and work/voluntary activities. It scores from 0 to 400, and a higher score indicates a higher activity level. Time points: Baseline (*t*_0_), 6-month post-surgery (*t*_5_), 12-month post-surgery (*t*_6_)

The *activPAL activity logger* and accompanying software *PALanalysis* (PAL Technologies, 50 Richmond Street, Glasgow, Scotland) are used to measure physical activity. The activPAL-monitor is a small (23.5mm × 43.0mm × 5mm LHW) light weight (9.5 g) device that is worn on the midline of the anterior, upper third of the participant’s thigh. The activPAL uses position sensors and accelerometers to capture movement and activity. *The PALanalysis* software (PAL Technologies, 50 Richmond Street, Glasgow, Scotland) will be used to analyse data on time spent in various activity levels, the number of steps taken, sit-to-stand transitions, and time spent sedentary or sleeping. The device is worn during the pre-operative period (*t*_0_ to *t*_1_). For the intervention group, the activPAL data is collected by the PT at an in-home visit and remotely transferred, using the PAL technologies provided app PALtransfer, to MA or JD for analysis and compilation of the feedback template.

- Mortality

Mortality data will be collected from the patient records.

Time points: 30 days (*t*_4_), 12 months (*t*_6_), 24 months (*t*_7_), and 36 months (*t*_8_) post-surgery.

#### Baseline variables and other outcomes

- Anthropometrics

Body weight (in kilogrammes) and body height (in centimetres) will be collected and presented as mean and SD. Time-point: Baseline (*t*_0_).

- Education level

A study-specific questionnaire will be used to collect data on education level (five levels ranging from compulsory school to university degree). Time-point: baseline (*t*_0_).

- Comorbidities

Charlson Comorbidity Index [47] will be used to quantify the burden of comorbid conditions. The occurrence of any of 19 different comorbid conditions is assigned a weight (ranging between 1 and 6), and the resulting metric is the sum of weights. Higher weights indicate a higher risk of mortality and more severe comorbidity. Results are presented as medians and proportions. Time-point: baseline (*t*_0_).

- Current medications

Medical journals and interviews will be used to collect data on current medications. Time-point: baseline (*t*_0_).

- Smoking and alcohol habits

A study-specific questionnaire will be used to collect information on smoking habits and alcohol use. Smoking will be assessed by interview and classified into three levels: *current smoker*, *previous smoker*, and *never smoker*. Alcohol habits will be assessed by the question “How many glasses of alcoholic beverages do you consume every week?” and answers are categorised into five levels (none to >14/week). Time-point: baseline (*t*_0_).

- Nutritional status and risk of malnutrition

Collected by interview, three areas will be used to assess nutritional status: *current underweight* (BMI<20 for patients under the age of 70, BMI <22 in patients over the age of 70), the occurrence of *unintentional weight loss* (yes or no), *perceived difficulties eating* (yes or no) [48]. If any of these three areas are fulfilled (low BMI or answered yes), the risk of malnutrition is considered heightened. Time-point: baseline (*t*_0_).

- Cognitive impairment

The Swedish version of the Mini-Mental Test Examination (MMSE-SR) [49] assesses cognitive impairment. Higher scores indicate better cognitive status (maximum 30 points). A cut-off of <24 points is indicative of cognitive impairment. Time-point: baseline (*t*_0_).

- Patient-centred goals of exercises

The PSFS-scale [33] is used in the intervention group to capture a minimum of one and a maximum of three situations/activities that they perceive as important in their lives but challenging to perform due to their medical condition. PTs design exercise regimens to improve chosen activities. The outcome is the specific activity (1 to 3) and grading of how difficult it is to perform (1 to 10). Time points: (*t*_0_ to *t*_1_).

### Participant timeline {13}

The participant timeline is shown in Table 1.

### Sample size {14}

The sample size was calculated based on the primary hypothesis that the intervention would (1) result in better physical performance measured by the 6MWT pre-surgery and (2) result in 1 grade lower post-operative complications according to CD classification 30 days post-surgery. The standard deviation (SD) used in the sample size calculations were based on data reported in a previous study [10]. For both outcomes, the calculations are based on the assumptions of equal group size and calculated for the independent t-test.

#### Power calculation for 6MWT

For the 6MWT, an SD of 111.5 m was assumed based on a previous study. A clinically important difference of 50 m was used in the calculations. To be able to detect a difference of 50 m, with an alpha level of 5% and a power of 0.80, a total sample size of 160 participants would be needed.

#### Power calculation for CD-classification

For the outcome of post-operative complications, an SD of 1.32 was assumed for the CD scale. A “pitman efficiency factor” was applied by dividing the estimated sample size by 0.864 to adjust for the CD classification as an ordinal scale. We assumed a difference of 1 grade between groups to be of clinical importance. With an alpha level of 5% and a power of 0.80, a total sample size of 58 would be needed. With the application of the correction factor (dividing the total sample size by 0.864), the final sample size needed is 67 evaluable participants.

### Recruitment {15}

Prior to this RCT, a feasibility study was performed [41]. The recruitment rate in the feasibility study was low (35% of eligible participants were recruited). A common reason for declining participation was the need for an extra visit to the hospital for the baseline visit (28%). To counteract this, we now offer taxi transportation to and from pre-operative visits (*t*_−1_ and *t*_1_). Another reason for declining participation was not wanting to delay the time to surgery (14%). The present study places more emphasis on the patient’s clinician informing the participant that no increase in risk is expected in the case of needing to postpone the surgery date for 1–2 weeks to allow for the intervention. More recruitment sites are used in the present study (4 vs 1) than in the feasibility study to lower the total time needed for completion of enrolment.

## Assignment of interventions: allocation

### Sequence generation {16a}

Computer-generated random number tables were constructed using free software, available online (http://www.jerrydallal.com/random/random_block_size.htm) and used for group allocation. Block randomisation with varying block sizes (*n*= 2, 4, or 6) with random ordering is applied to minimise the possibility of predicting group allocation.

### Concealment mechanism {16b}

The allocation table will be uploaded into an electronic database (REDCap), and randomisation will be performed using the randomising function of the database. Full access to the allocation table in the database is restricted to ER and MA, whereas for all other persons involved in the study, the allocation sequence will be concealed.

### Implementation {16c}

MA generated the random allocation table using free software available online (http://www.jerrydallal.com/random/random_block_size.htm). Enrolment of participants is conducted by site-personal (nurses or PTs) whom first approach potential participants identified from a list of patients referred to the hospital for diagnosis and possible surgical treatment. If potential participants match the inclusion criteria, informed consent is obtained by study personnel (nurses or PTs) prior to baseline testing. After the first baseline visit (*t*_0_), participants are randomised and allocated using the randomisation function of the electronic database of the study that ensures the concealment of the allocation order. Randomisation is performed by ER, MA, JD, or ST).

## Assignment of interventions: blinding

### Who will be blinded {17a}

Outcome assessors are blinded. Due to the nature of the intervention (exercise), neither care providers nor participants could be blinded. Participants are instructed not to reveal their assignment to assessors at the follow-ups (*t*_−1_, *t*_2_, *t*_3_).

### Procedure for unblinding if needed {17b}

Not applicable.

## Data collection and management

### Plans for assessment and collection of outcomes {18a}

#### Training of assessors

Before the start of enrolment, all study personnel involved in testing (PTs, nurses, research nurses) or delivery of the intervention (PTs) received training in all methods to be used in the study from ER, JD, and MA. Written instructions and checklists for the flow of participants through the study were developed and delivered to study personnel in printed documents and digital forms. A digital communication platform between involved personnel was established using Microsoft Teams (MS-Teams) (Microsoft Corporation, One Microsoft Way, Redmond, WA 98052-7329, USA). Access to specific MS-Teams areas (and associated files) was restricted and based on a need-to-know basis. All data collection instruments were provided with written instructions and video recordings or slide shows with instructions for specific methods such as the IMT-training device. During the training of physiotherapists before the first inclusion, workshops were held (digitally) where all protocols, tests, questionnaires, and related procedures were discussed. Between workshops, physiotherapists also completed a task where data for a hypothetical participant was to be registered into the protocols according to the procedures in the study. The assignment was discussed in groups at the follow-up workshop, and any questions or unclarity regarding the protocols or procedures were discussed and resolved. The digital platform is available through the study course to both promote learning of procedures at the beginning of the study and prevent procedural drift over time.

### Plans to promote participant retention and complete follow-up {18b}

An intention-to-treat approach will be used. Thus, all randomised participants will be considered to have received the treatment to which they were allocated, even if they choose to discontinue their participation or have low compliance with the treatment. Outcome data available from medical records (post-operative complications) will be collected as planned unless participants do not explicitly state that this should not be done.

### Data management {19}

Data collected in written protocols or checklists will be stored in a secure place (locked file storage or equivalent) and later transferred into an electronic data storage using REDCap electronic data capture tools hosted at Karolinska Institute [50, 51]. REDCap is a secure, web-based software platform designed to support data capture for research studies, providing (1) an intuitive interface for validated data capture; (2) audit trails for tracking data manipulation and export procedures; (3) automated export procedures for seamless data downloads to common statistical packages; and (4) procedures for data integration and interoperability with external sources.

### Confidentiality {27}

All personal data collected will be handled according to EU data protection rules described in the General Data Protection Regulation (GDPR).

### Plans for collection, laboratory evaluation, and storage of biological specimens for genetic or molecular analysis in this trial/future use {33}

Not applicable.

## Statistical methods

### Statistical methods for primary and secondary outcomes {20a}

An intention-to-treat approach will be used and supplemented with a per-protocol analysis. Primary and secondary outcomes will be compared between groups. Data will be examined regarding normality, missing data, and outliers.

The hypothesis is that prehabilitation will result in better physical function in the pre-operative phase and fewer complications in the post-operative phase. This will be tested against the null hypothesis, i.e. prehabilitation will result in similar walking distance and complications as in the usual care group. An analysis of covariance (ANCOVA) will be performed to assess change in pre-operative physical function between groups (first primary outcome). Ordinal logistic regression will be used to analyse the differences between groups in post-operative complications (second primary outcome).

Demographics for the study population will be presented using descriptive statistics (mean and SD, median with IQR or min-max, and numbers with proportions) depending on the type of data collected and its distribution. Adherence to supervised exercise sessions will be presented using descriptive statistics (percentage of attended out of planned supervised sessions). Furthermore, all primary and secondary outcomes will be presented using these descriptive statistics at all time points. Differences between groups at single time points will be analysed by unpaired *t*-test, Mann-Whitney-test, chi-squared-test, or Fisher’s exact test, depending on the data type and distribution. General linear mixed models (GLMM) will be used to assess differences between groups over several repeated measurements, adjusting for relevant covariates. Point estimates and test statistics will be reported with the two-sided *p*-value and complemented with 95% confidence intervals to describe the direction and magnitude of the effect.

### Interim analyses {21b}

No interim analysis will be performed.

### Methods for additional analyses (e.g. subgroup analyses) {20b}

Sub-group analyses based on surgical procedure (open or minimally invasive) are planned.

### Methods in analysis to handle protocol non-adherence and any statistical methods to handle missing data {20c}

All participants will be analysed within the group to which they are randomised. Missing data will be handled by imputation, with the exact method depending on the type of missingness [52].

### Plans to give access to the full protocol, participant-level data, and statistical code {31c}

As long as data is pseudo-anonymized, requests for access to the data can be put to our Research Data Office (rdo@ki.se) at Karolinska Institutet or via the Swedish National Data service catalogue and will be handled according to the relevant legislation. This will require a data transfer agreement or similar with the recipient of the data. Meta-level data will be made available on the website Swedish National Data service (https://snd.gu.se/en). The statistical codes will be made available in future publications in open-access journals; this will enhance transparency and open science.

## Oversight and monitoring

### Composition of the coordinating Centre and trial steering committee {5d}

#### Principal investigator assumes the following role and responsibilities

- Design and conduct of CANOPTIPHYS
- Preparations of protocols and revisions
- Organising meetings between Trial Management Group (TMG) and lead investigators
- Publication of study reports

#### Trial management group (TMG)

The TMG (ER, MA, JD, ST) coordinates all day-to-day operations regarding the study. All participating staff has direct access to the TMG through phone or digital communications for guidance on operational questions. The TMG organises meetings with participating centres (both hospital sites responsible for recruiting participants as well as the primary health care centres) one to two times every semester.

#### Lead investigators

At each participating centre, a lead investigator (a senior surgeon) will be appointed and take responsibility for the recruitment and identification of participants at each site. Any changes or amendments to the protocol will be communicated to the lead investigator for approval before being implemented.

### Composition of the data monitoring committee, its role, and reporting structure {21a}

No data monitoring committee is appointed. The single-blinding of the study (assessors are blinded) ensures that the PI and lead investigators will assume the role of following up on any adverse events occurring in the trial since they will have unblinded access to data on the participants of the trial.

### Adverse event reporting and harms {22}

AEs are collected by PTs and collected in written records. Suspected AEs are reported to the physician responsible for the participants’ cancer care and the lead investigator at the site.

### Frequency and plans for auditing trial conduct {23}

No auditing is planned since the study does not involve any pharmacological treatment. All decisions regarding the trial or amendments or alterations of material will be documented in an electronic logbook (ELN) at Karolinska Institutet, enabling an electronic audit trail of the study. The PI maintains the logbook. The process is independent of sponsors.

### Plans for communicating important protocol amendments to relevant parties (e.g. trial participants, ethical committees) {25}

Relevant changes or protocol amendments will be submitted to the ethical review board for approval before implementation. Lead investigators at recruitment sites and site personnel will be contacted by the TMG and informed of any changes to the study protocol or materials used.

The PI will submit relevant changes to trial registries.

## Dissemination plans {31a}

Results will be published in peer-reviewed journals and communicated at scientific meetings. Trial participants are informed that they can contact the PI if they wish to have access to their individual data and/or results generated in the trial.

## Discussion

The study will start recruiting participants during an ongoing pandemic of SARS-Cov-2 with the widespread transmission of the virus in the community. The ramifications of the pandemic on inclusion rate, dropout rate, or other operational issues for involved organisations are challenging to foresee. We believe there is a risk of slow inclusion rates due to the fear of contracting viruses from study personnel entering the home of participants. Waiting time might be altered (shortened or prolonged) due to increased demand for hospital beds and a possible shortage of staff during pandemic peaks. Since surgery of malignant tumours is of high priority in the healthcare system, an effect of the pandemic might be that non-life-threatening conditions will be postponed and surgery due to malignancies prioritised. This might render the waiting times for surgery shorter than expected and shorter than needed to allow for the 14 days of pre-operative optimisation required in this study.

Although trying to maximise geographical coverage of the Stockholm city area, we realise that there will be instances where includable subjects are lost due to lack of coverage from rehabilitation units.

We have chosen to focus the current study on the physical aspects of prehabilitation even though other potential areas of prehabilitation are possible, such as nutritional support (in under- or overweight subjects), supplementation of proteins, or psychological support. These aspects are monitored and provided to patients within the usual care pathways.

## Trial status

Recruitment started in March of 2021 and will approximately end in June 2023.

## Data Availability

Access to the final dataset will be restricted to the PI (ER) and the Research Data Office (RDO) at Karolinska Institute. Inquiries and requests for access to the dataset will be handled by the RDO at Karolinska Institute.

## Abbreviations

AE: Adverse event
ANCOVA: Analysis of covariance
CCI: Comprehensive Complication Index
CD: Clavien-Dindo classification system for surgical complications
CRC: Colorectal cancer
ELN: Electronic logbook for research projects at Karolinska Institutet
GLMM: General linear mixed model
IMT: Inspiratory muscle training
IQR: Interquartile range
KI: Karolinska Institutet
PC: Post-operative complications
PI: Principal Investigator
PRP: Post-operative Recovery Profile
PSFS: Patient-Specific Functional Scale
PT: Physiotherapist
RDO: Research Data Office
SCP: Standardised Cancer care Pathways
SD: Standard deviation
TMG: Trial Management Group
6MWT: 6-minute walk test
